# Disturbances in branched-chain amino acid profile and poor daily functioning in mildly depressed chronic obstructive pulmonary disease patients

**DOI:** 10.1186/s12890-021-01719-9

**Published:** 2021-11-07

**Authors:** Marisa R. Pinson, Nicolaas E. P. Deutz, Rajesh Harrykissoon, Anthony J. Zachria, Mariëlle P. K. J. Engelen

**Affiliations:** 1 Department of Health and Kinesiology, Center for Translational Research in Aging and Longevity, Texas A&M University, College Station, TX USA; 2 Department of Neuroscience and Experimental Therapeutics, Texas A&M University Health Science Center, Bryan, TX USA; 3 Pulmonary, Critical Care and Sleep Medicine, Scott and White Medical Center, College Station, TX USA

**Keywords:** COPD, Depression, Branched-chain amino acids, Physical performance

## Abstract

**Background:**

Depression is one of the most common and untreated comorbidities in chronic obstructive pulmonary disease (COPD), and is associated with poor health outcomes (e.g. increased hospitalization/exacerbation rates). Although metabolic disturbances have been suggested in depressed non-diseased conditions, comprehensive metabolic phenotyping has never been conducted in those with COPD. We examined whether depressed COPD patients have certain clinical/functional features and exhibit a specific amino acid phenotype which may guide the development of targeted (nutritional) therapies.

**Methods:**

Seventy-eight outpatients with moderate to severe COPD (GOLD II–IV) were stratified based on presence of depression using a validated questionnaire. Lung function, disease history, habitual physical activity and protein intake, body composition, cognitive and physical performance, and quality of life were measured. Comprehensive metabolic flux analysis was conducted by pulse stable amino acid isotope administration. We obtained blood samples to measure postabsorptive kinetics (production and clearance rates) and plasma concentrations of amino acids by LC–MS/MS. Data are expressed as mean [95% CI]. Stats were done by graphpad Prism 9.1.0. ɑ < 0.05.

**Results:**

The COPD depressed (CD, n = 27) patients on average had mild depression, were obese (BMI: 31.7 [28.4, 34.9] kg/m^2^), and were characterized by shorter 6-min walk distance (*P* = 0.055), physical inactivity (*P* = 0.03), and poor quality of life (*P* = 0.01) compared to the non-depressed COPD (CN, n = 51) group. Lung function, disease history, body composition, cognitive performance, and daily protein intake were not different between the groups. In the CD group, plasma branched chain amino acid concentration (BCAA) was lower (*P* = 0.02), whereas leucine (*P* = 0.01) and phenylalanine (*P* = 0.003) clearance rates were higher. Reduced values were found for tyrosine plasma concentration (*P* = 0.005) even after adjustment for the large neutral amino acid concentration (= sum BCAA, tyrosine, phenylalanine and tryptophan) as a marker of dopamine synthesis (*P* = 0.048).

**Conclusion:**

Mild depression in COPD is associated with poor daily performance and quality of life, and a set of metabolic changes in depressed COPD that include perturbation of large neutral amino acids, specifically the BCAAs.

*Trial registration* clinicaltrials.gov: NCT01787682, 11 February 2013—Retrospectively registered; NCT02770092, 12 May 2016—Retrospectively registered; NCT02780219, 23 May 2016—Retrospectively registered; NCT03796455, 8 January 2019—Retrospectively registered.

**Supplementary Information:**

The online version contains supplementary material available at 10.1186/s12890-021-01719-9.

## Background

Amongst the three chronic conditions that affect 60 million people in the USA (diabetes, heart disease, and chronic obstructive pulmonary disease (COPD)), patients with COPD have the highest prevalence of depression. Depression is one of the most common and untreated comorbidities in COPD [1], affecting 16–71% of patients with moderate to severe COPD [2–6]. Even conservatively, this is nearly twice the average for the U.S. population as a whole (7.1% prevalence in 2017) [7].

Depression in COPD is associated with reduced quality of life (QoL), worse treatment compliance, and higher rates of acute exacerbations [8–11]. As a result, COPD patients with depression are more likely to have an emergency room visit (48%) and hospitalization (60%) [8], and have significantly more relapses [12]. Frequent exacerbations, on the other hand, give rise to a poor QoL, resulting in a vicious circle. Conflicting evidence exists whether changes in body composition may influence depression status in COPD [9, 13–18] and remains to be clarified as additional studies are done.

### Physical function and cognition

COPD patients with higher levels of depressive symptoms report worse daily physical functioning [19, 20] and have lower functional capacity (e.g., 6 min walk test, incremental tests) [21–24]. These patients are more likely to use oxygen during the test [9, 16, 25]. Additionally, other studies linked depression to skeletal muscle weakness [26] and decreased physical activity [13]. While many have shown a link between depression and cognitive impairment in the general population, studies focused on COPD patients have not yet definitively shown such a link [13, 27, 28].

### Metabolic phenotype

Although COPD is associated with amino acid metabolic deregulations [29–34], limited research has been conducted examining the role of amino acid perturbations as potential metabolic mechanisms underlying depression in COPD. Impaired plasma levels of tryptophan, a precursor of serotonin, have previously been observed in depressed patients (without COPD) as reflected by a reduced ratio of tryptophan to large neutral amino acids (LNAA which include tyrosine, phenylalanine, and the branched-chain amino acids (BCAA)). When depression scores improved in depressed patients, there was a corresponding increase in tryptophan/LNAA ratio, meaning more tryptophan can cross the blood brain barrier with less competition for the transporter [35]. The brain tryptophan concentration was found to be related to the cerebral serotonin synthesis [36]. In addition, decreased levels of BCAAs have previously been identified in COPD patients and particularly in those with muscle loss [29, 37], potentially contributing to increased risk for depression. Furthermore, decreased bioavailability of arginine has been observed in major depressive disorder [38, 39] and altered arginine metabolism has been identified in COPD [33], suggesting an additional pathway by which altered amino acid metabolism may contribute to depression in COPD patients.

To identify whether depression in COPD is associated with a specific metabolic phenotype, we examined in the present study whether higher depression scores were associated with specific changes in plasma amino acid profile and whole-body production rates of amino acids known to play a role in mood (e.g. LNAA, BCAA, and arginine). COPD patients were recruited from the MEDIT trial (MEtabolism of Disease with Isotope Tracers). As multiple other factors may also affect amino acid metabolism, the subjects were well characterized by general- and disease specific features (e.g. lung function, disease history, comorbidity index, habitual dietary intake, physical activity level), body composition, muscle and cognitive function. Identification of a metabolic phenotype in a depressed COPD population may help to gain insight into the underlying pathophysiological mechanisms contributing to increased risk for depression, guiding a more targeted antidepressant treatment and revealing potential targets for nutritional intervention.

## Materials and methods

### Subjects

Subjects with COPD were recruited from the MEDIT (MEtabolism of Disease with Isotope Tracers) trial, a large controlled trial in healthy and diseased subjects. Patients with clinically stable COPD under the routine management of the pulmonary clinics were recruited. COPD subjects were further subdivided into non-depressed with COPD and depressed with COPD based on recorded Hospital Anxiety and Depression Scale depression subsection score (a score of 8 or higher was used to define clinically relevant depression) [10, 40–42]. In the non-depressed with COPD group, only subjects with a depression score of less than 8 were included (non-depressed). Groups controlled for age by selecting subjects to have comparable age ranges between groups. In total, 178 subjects were assessed for eligibility, 27 depressed COPD (CD group) and 51 non-depressed COPD (CN group) subjects were included for data analysis. The inclusion criteria for all groups were age 46 to 76 years old in order to control for variances due to age, and the ability to walk, sit, and stand independently. All COPD patients were clinically stable with no history of exacerbation of their disease or a respiratory tract infection at least 4 weeks prior to the study. As previously described, the exclusion criteria were the presence of a fever within 3 days prior to the study day, acute illness, a metabolically unstable chronic illness, pre-existing untreated metabolic or renal disease, malignancy, recent surgery, and use of oral corticosteroids 1 month prior to the study because of known effects on amino acid metabolism [43, 44]. As part of the screening process, a medical history was conducted which included the number of exacerbations and medication used. Study days started in the morning under postabsorptive conditions (no food after 12 a.m.) and lasted 5 h.

All subjects were studied at the Clinical Research Unit of the Center for Translational Research in Aging and Longevity, housed at the Human Clinical Research Building, Texas A&M University. Before any measurements were performed, written informed consent was obtained from all subjects. The study was conducted in accordance with the Declaration of Helsinki, and the protocol was approved by the Institutional Review Board of Texas A&M University before being registered on ClinicalTrials.gov (NCT01787682, NCT02770092, NCT02780219, NCT03796455).

### Diet, physical activity, COPD severity, comorbidity assessment, and quality of life questionnaires

Habitual dietary intake was assessed by 24-h dietary recall, and habitual physical activity level by Physical Activity Scale for the Elderly Questionnaire (PASE) [45]. The COPD Assessment Test (CAT) [46] was performed to assess the level of dyspnea, and Charlson index for assessment of associated comorbidities [47]. The category of Cardiovascular Disease is composed of hypertension, congestive heart failure, myocardial infarction, peripheral vascular disease, cerebrovascular disease, pulmonary circulation disorders, and dyslipidemia. Profile of Mood States (POMS) Questionnaire was used to quantify negative and total mood states [48]. St. George Respiratory Questionnaire (SGRQ) was used to assess disease specific quality of life [49]. A change in the total score for the SGRQ that represents a clinically important difference is 4 units [50].

### Anthropometrics, body composition, and lung function

All study procedures were identical in both groups and conducted as previously described [44]. Briefly, a digital beam scale and stadiometer were used to measure body weight and height. Blood pressure was measured on the upper arm after a 5 min rest sitting. Whole body, trunk and extremity (arms and legs) fat mass (FM), and fat-free mass (FFM) were obtained from all subjects while in a supine position by dual-energy X-ray absorptiometry (Hologic QDR 4500/Version 12.7.3.1 (Bedford, MA)). Anthropometric and body composition were standardized for height (kg/m^2^) [51] to obtain the body mass index (BMI, kg/m^2^), fat free mass index (FFMI), fat mass index (FMI), and the appendicular skeletal muscle index (ASMI). Spirometry was performed using the Microloop Peak flow Meter (CareFusion, San Diego, CA). Forced expiratory volume in 1 s (FEV1) and forced vital capacity (FVC) was measured in all participants, with the highest value from ≥ 3 technically acceptable maneuvers being used [52]. Transcutaneous oxygen saturation was measured using pulse oximetry.

### Physical function

Markers of physical function included assessment of upper and lower limb skeletal muscle function, gait speed, and the 6-min walk test (6MWT) and were conducted as done previously [44]. Briefly, leg strength was quantified by using a Kincom isokinetic dynamometry (Isokinetic International, Chattanooga, TN, USA) to measure the peak leg force during one leg reciprocal extensions (at 60°/s) [53, 54]. Handgrip strength was quantified by using a Vernier dynamometry (Vernier software and Technology, Beaverton, OR, USA) to measure the peak handgrip force that the subject was able to generate out of 3 reproducible repetitions, with 1 min of rest between each attempt [53]. Leg endurance was assessed by performing 30 maximal single leg extensions (at 120°/s) to assess a decrease in leg force output [54]. The average force output of the last 3 repetitions as percentage of the first 3 repetitions was used (e.g. (avg first 3 − avg last 3)/avg first 3). 6MWT was performed as per American Thoracic Society standards [55]. Subjects had access to their walking aid and/or oxygen if required.

### Neurocognitive function

Subjects completed the Trail Making Test (TMT) [56] and Stroop color-word tests (SCWTs) [57], which are known to be simple and sensitive in assessing neurocognitive impairment. TMT consists of two subtasks, Part A and Part B. SCWTs consists of three subtasks (I, II, and III), and the interference score was calculated [58, 59].

### Blood analysis to assess markers of metabolic/clinical health

Arterialized-venous blood was put in Li-heparinized or EDTA tubes (Becton Dickinson Vacutainer system, Franklin Lakes, New Jersey, USA), immediately put on ice to minimize enzymatic reactions and was centrifuged at 4 °C, 3120 × g for 5 min to isolate plasma. A part of the plasma was aliquoted into tubes with 0.1 vol of 33% (w/w) trichloroacetic and then vortexed for the denaturation of proteins. Samples were immediately frozen and stored at − 80 °C for later processing. Plasma amino acid concentrations of the essential amino acids, tryptophan, large neutral amino acids (LNAA), and the branched-chain amino acids (BCAA) were analyzed batch-wise by LC–MS/MS by isotope dilution, as previously reported [60]. High-sensitivity C-reactive protein (hs-CRP) was measured using a particle enhanced immuno-turbidimetric assay, and fasting glucose concentration was measured using a hexokinase method (Cobas c111, Roche Diagnostics, Mannheim, Germany). Homeostatic model assessment (HOMA) index was calculated to assess β-cell function and insulin resistance [61].

### Stable tracer infusion by IV pulse administration

As previously described [60], a peripheral line was placed in a vein of the lower arm for stable tracer infusions and in a superficial dorsal vein of the contralateral hand for blood sampling. A venous blood sample was initially collected to measure baseline enrichment. Then an IV pulse containing a cocktail of amino acid stable tracers (Cambridge Isotope Laboratories: Woburn, MA, USA) was administered, as described previously [60]. Arterialized-venous blood was sampled at multiple time points (t = 10, 20, 30, 60, and 120 min) for 2 h after pulse administration.

Whole-body production (WBP) rates were calculated with non-compartmental analysis using GraphPad Prism (Version 9.2.0). WBP for all amino acids was calculated as tracer pulse dose/area under the curve (AUC) from t = 10 to t = 120 min [62]. AUC was calculated by fitting the decay of the TTR of the stable tracers that were injected with the pulse with either two exponentials: TTR (t) = a * exp(− k1 * t) + b * exp(− k2 * t) or three exponentials: TTR (t) = a * exp(− k1 * t) + b * exp(− k2 * t) + c * exp(− k3 * t) [63]. Conversion of one amino acid into another amino acid was done by using the WBP of the product amino acid and AUC of the TTR from pulse of the product/substrate [60]. Clearance of the stable tracers was calculated as WBP/plasma concentration [64].

### Statistical analysis

All results were expressed as means [95% CI] or geometric means [95% CI] when data was log-transformed. The normality of the data was tested by the D’Agostino–Pearson omnibus normality test. The Robust regression and Outlier removal (ROUT) test (Q = 5%) were performed on normal data to identify outliers. An one-way ANCOVA was used to compare the groups (CN vs. CD). Covariates used in all analyses included BMI and age, as these are known to influence amino acid metabolism and did change our findings when included as covariates. We checked to see if including antidepressant use, SSRI use, SNRI use, anxiolytic use, sex, FEV1, smoking status, CRP, carbohydrate intake, and comorbidities as a covariate altered ANCOVA results and observed no difference (data not shown), so therefore did not include these variables as a covariate in analyses. When the normality test failed, data was transformed using natural log to normalize and then analyzed when normality was confirmed. The statistical packages within GraphPad Prism (GraphPad Software, La Jolla, CA, Version 9) were used for data analysis. The significance level was set at q value (< 0.05) which is the Benjamini–Hochberg FDR corrected *P* value.

## Results

### Demographics, clinical characteristics, and comorbidities

**Fig. 1:**
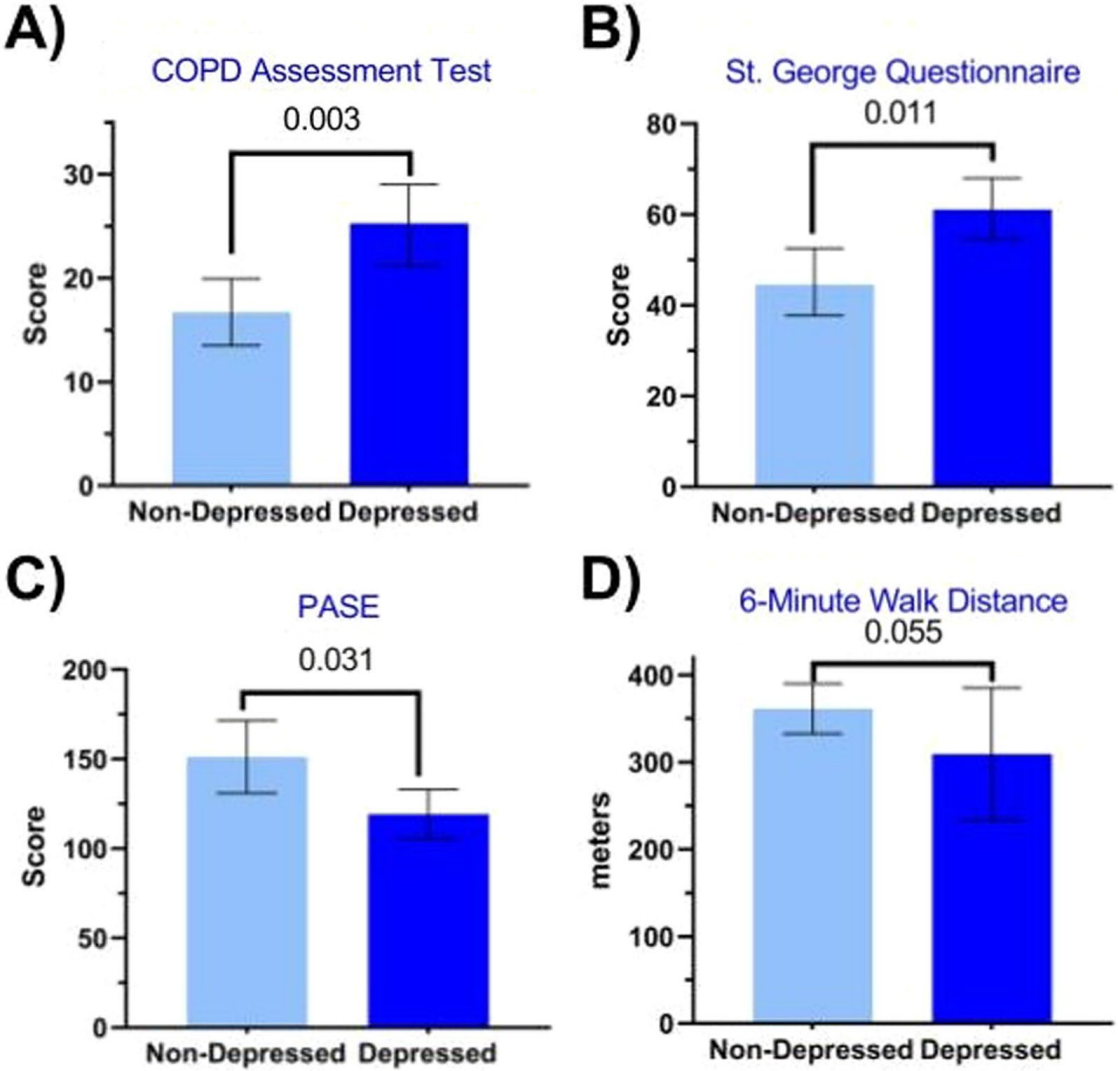
Quality of life and physical function in non-depressed and depressed COPD patients. **A** COPD assessment test measuring impact of COPD on patient’s health. **B** Total score of St. George Questionnaire including Symptoms, Activities, and Impacts. **C** Physical Activity Scale for the Elderly (PASE). **D** Total distance traveled during 6-Min Walk Test. Mean ± 95% CI by ANCOVA with age and BMI as covariates

The depressed COPD group (CD) was characterized by mild depression (depression scores: 9.185 [8.545, 9.825]) and had on average a BMI > 30 kg/m^2^ (BMI: 31.65 [28.40, 34.90] kg/m^2^), which is classifiable as obese (Table 1). In contrast, the non-depressed COPD (CN) on average had a BMI < 30 kg/m^2^ (BMI: 29.08 [27.30, 30.85] kg/m^2^), though this was not statistically different from CD. The CD (n = 27) and CN (n = 51) groups were of a similar age range (46–76 years) and had comparable lung function, O_2_% saturation, O_2_ use, and GOLD score distribution (1: 9.8% CN v. 7.4% CD, 2: 29.4% v. 25.9%, 3: 33.3% v. 40.7%, 4: 27.5% v. 25.9%). While the CD and CN groups had comparable COPD severity, dyspnea symptoms and disease history (exacerbation and hospitalization frequency in preceding year), the CD group had higher CAT scores (*P* = 0.003; Fig. 1A and Table 1), indicating a higher COPD impact on their life. Systolic blood pressure (*P* = 0.036) and CRP (*P* = 0.007), as a marker of systemic inflammation, were lower in CD but no differences were found in insulin or glucose plasma concentrations, between the groups (Table 2). Although medication use and Charlson comorbidity index were comparable between the groups, a higher rate of sleep disordered breathing was observed in the CD group (13.7% CN v. 44.4% CD, *P* = 0.005) (Table 1). Additionally, there was a higher prevalence in CD of sleep apnea (37.3% v. 55.6%) and cardiovascular disease (62.7% CN v. 74.1% CD) though both were not significant. Furthermore, there was no difference in prevalence of self-reported psychological issues (41.2% CN v. 48.1% CD) and no difference in the proportion of patients using antidepressants/anxiolytics between non-depressed COPD and those experiencing depression (48% CN vs. 59% CD).

**Table 1:** Demographics, clinical characteristics, and comorbidities of the non-depressed and depressed COPD groups

	COPD non-depressed (CN) (n = 51)	COPD depressed (CD) (n = 27)	Estimated difference	Unpaired t-test *p value*
Demographics				
Age (years)	64.72 [63.16, 66.28]	64.04 [57.90, 69.64]	− 0.68 [− 3.60, 2.24]	0.643
Sex (male/female)	26/25	13/14		> 0.999
Level of education (score)	2.19 [1.56, 2.81]	2.00 [1.39, 2.62]	− 0.19 [− 1.15, 0.78]	0.698
General characteristics				
Body weight (kg)	79.03 [73.15, 85.38]	83.96 [74.03, 95.23]	5.76 [− 4.93, 16.45]	*0.384*
Height (m)	1.67 [1.64, 1.70]	1.66 [1.63, 1.69]	− 0.01 [− 0.05, 0.03]	*0.667*
Body Mass Index (kg/m^2^)	29.08 [27.30, 30.85]	31.65 [28.40, 34.90]	2.574 [− 0.75, 5.90]	0.128
COPD parameters				
FEV1 (% of predicted)	43.86 [38.94, 48.79]	44.59 [37.13, 52.06]	− 1.66 [− 9.01, 5.69]	*0.911*
GOLD stage (I/II/III/IV)	5/15/17/14	2/7/11/7		0.765
Years of COPD related symptom (yr)	11.81 [8.74, 14.88]	10.21 [7.18, 13.24]	− 1.51 [− 51.87, 48.86]	*0.644*
No. of hospitalizations past year for exacerbation	0.24 [0.07, 0.42]	0.42 [0.12, 0.73]	0.26 [− 0.06, 0.57]	0.267
No. of exacerbations in the past year	0.57 [0.25, 0.89]	1.00 [0.29, 1.71]	0.56 [− 0.12, 1.24]	0.203
O2 use (no/yes)	30/21	11/16		0.157
mMRC dyspnea grade	2.20 [1.92, 2.49]	2.48 [2.14, 2.82]	0.32 [− 0.15, 0.79]	0.238
CAT score	16.71 [13.48, 19.94]	25.27 [21.16, 29.38]	9.04 [3.47, 14.61]	**0.003**
Current smoker (yes/no)	29/21	18/8		0.456
Years smoking	34.57 [30.00, 40.00]	34.50 [30.00, 40.00]	1.57 [− 3.76, 6.91]	0.619
Smoke pack years	46.31 [32.00, 45.00]	48.96 [25.00, 66.00]	1.74 [− 13.19, 16.65]	*0.716*
Vitals				
Heart rate (bpm)	70.90 [66.00, 73.00]	75.48 [67.00, 83.00]	5.02 [− 0.44, 10.47]	0.090
Systolic BP (mmHg)	137.20 [127.00, 141.00]	130.20 [117.00, 142.00]	− 8.81 [− 16.79, − 0.84]	*0.085*
Diastolic BP (mmHg)	78.41 [76.00, 82.00]	77.33 [71.00, 83.00]	− 1.78 [− 5.83, 2.28]	0.594
Temperature (°C)	36.52 [36.40, 36.67]	36.57 [36.17, 36.94]	0.07 [− 0.13, 0.26]	0.589
O2 saturation (%)	95.31 [94.00, 97.00]	94.81 [94.00, 97.00]	− 0.46 [− 2.014, 1.10]	*0.155*
Comorbidities				
Charlson comorbidity index (score)	1.80 [1.52, 2.09]	1.93 [1.58, 2.27]	0.30 [− 0.07, 0.67]	0.446
Cardiovascular disease (yes/no)	32/19	20/7		0.449
Diabetes (yes/no)	12/39	6/21		> 0.999
Glucose intolerance (yes/no)	5/46	2/25		> 0.999
Obstructive sleep apnea (yes/no)	19/32	15/12		0.153
Sleep disordered breathing (yes/no)	7/44	12/15		**0.005**
Osteoporosis (yes/no)	3/48	1/25		> 0.999
Other neurological disorders (yes/no)	6/45	5/22		0.499
Cognitive deficit (yes/no)	3/48	2/25		> 0.999
Psychological issues (yes/no)	21/30	13/14		0.634
Dementia (yes/no)	2/49	0/27		0.542
Medications				
Antianxiety or antidepressant agent (yes/no)	24/27	16/11		0.474
Anxiolytic agent (yes/no)	7/44	4/23		> 0.999
Antidepressant (yes/no)	23/28	15/12		0.477
*SSRI (yes/no)*	16/35	9/18		> 0.999
*SNRI (yes/no)*	10/41	3/24		0.525
Antipsychotic (yes/no)	5/45	4/23		0.712

Values are mean [95% CI], except for when data was log-transformed for which geometric mean [95% CI] was used. Education level: 0 = Associate, 1 = Bachelor, 2 = Master, 3 = Doctoral or professional degree. FEV1: Forced Expiratory Volume in 1 s. O2: supplemental oxygen, mMRC: modified medical research council dyspnea scale. Statistics are by unpaired t-test, normal text is untransformed data and italicized is log-transformed data, bold is *P* < 0.05

**Table 2:** Blood measurements of the non-depressed and depressed COPD groups

	COPD non-depressed (CN) (n = 51)	COPD depressed (CD) (n = 27)	Estimated difference	ANCOVA *p value*
Blood measurements				
CRP (mg/L)	6.81 [4.19, 9.42]	5.72 [1.77, 9.66]	− 2.27 [− 6.69, 2.15]	***0.007***
Glucose (mmol/L)	5.79 [5.54, 6.05]	5.74 [5.36, 6.12]	− 0.19 [− 0.61, 0.24]	*0.410*
Insulin (uIU/mL)	13.71 [9.30, 18.11]	12.99 [7.09, 18.88]	− 1.15 [− 8.34, 6.04]	*0.490*
HOMA Index	3.79 [2.44, 5.13]	3.28 [1.65, 4.92]	− 0.69 [− 2.13, 0.74]	*0.354*

Values are mean [95% CI], except for when data was log-transformed for which geometric mean [95% CI] was used. CRP: C-reactive protein. Homeostatic model assessment (HOMA) is a method for assessing β-cell function and insulin resistance. Statistics are by ANCOVA with age and BMI as covariates, normal text is untransformed data and italicized is log-transformed data, bold is *P* < 0.05

### Systemic health

#### Wellbeing, quality of life, and lifestyle

Besides mild depression, the CD group was characterized by higher anxiety scores (7.93 [6.59, 9.26], *P* = 0.002) (Additional file 1: Table S1) than the CN group. The CD group scored worse for quality of life as reflected by the St. George Questionnaire total score (ANCOVA, *P* = 0.011; Fig. 1B) and the subscores Activity (ANCOVA, *P* = 0.021), Impact (ANCOVA, *P* = 0.004) and Symptoms (ANCOVA, *P* = 0.065) subscores. Furthermore, the CD group was less physically active (ANCOVA, *P* = 0.031; Fig. 1C) and consumed more carbohydrates (*P* = 0.043; Additional file 1: Table S1).

#### Body composition and physical and cognitive function

Body composition was not different between the CD and the CN groups as reflected by comparable values for lean mass, fat mass, and visceral adipose tissue (Additional file 1: Table S2). There was a trend for lower 6MWT (ANCOVA, *P* = 0.055; Fig. 1D) in the CD group. Upper- and lower-limb muscle strength and endurance measurements and gait speed were not different (Additional file 1: Table S2). Although TMT and Stroop test scores were comparable between the CD and CN groups, the CD group was on average characterized by mild cognitive impairment as defined by Montreal Cognitive Assessment (MoCA) scores < 26 (Additional file 1: Table S2).

### Metabolic profiling

#### Large neutral amino acid metabolism

**Fig. 2:**
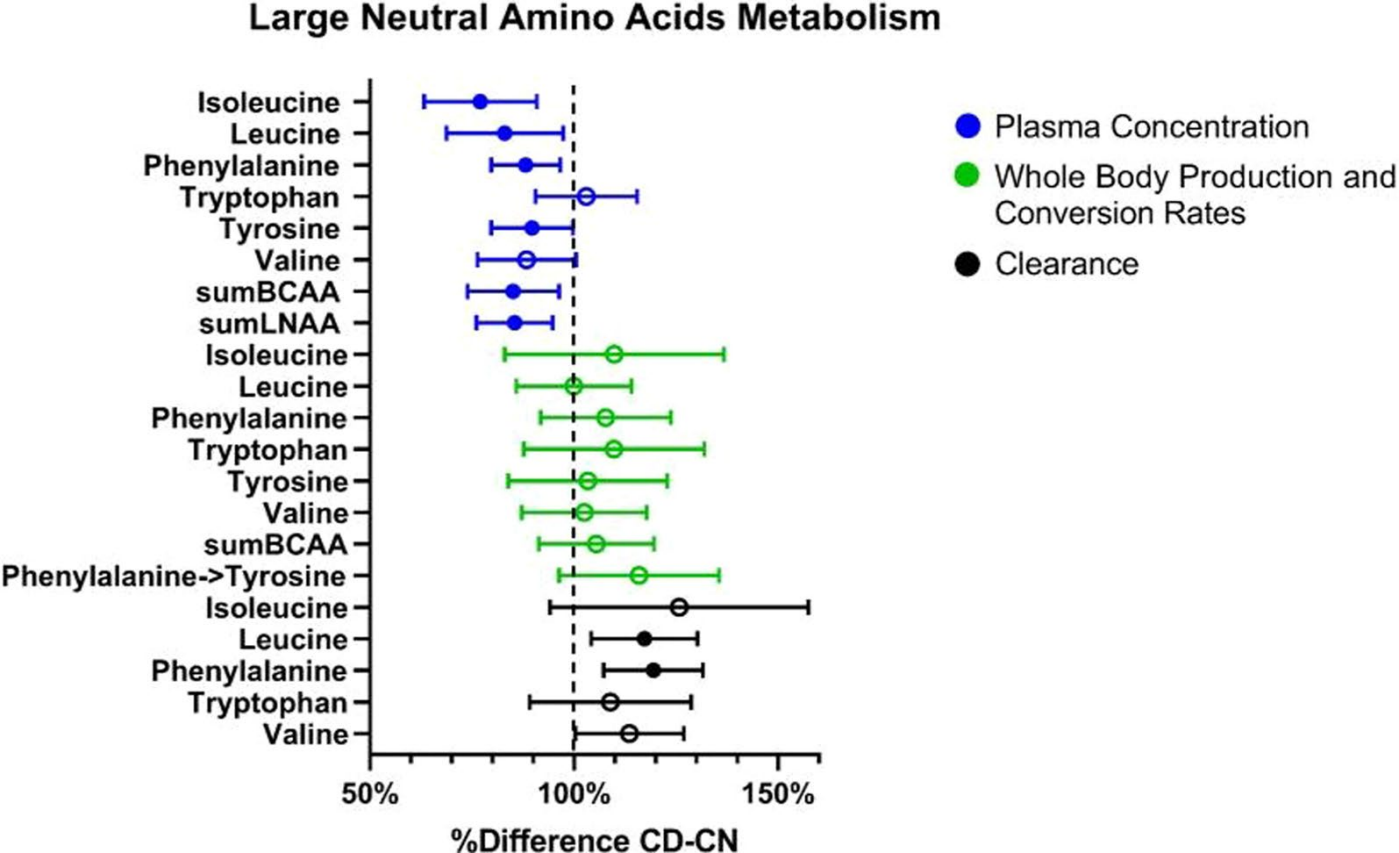
Differences in large neutral amino acid metabolism between non-depressed (CN) and depressed (CD) COPD patients. Percent difference of CD relative to CN. Mean ± 95% CI. Filled in circles are *P* < 0.05 by ANCOVA with age and BMI as covariates

The sum plasma concentration of the large neutral amino acids (sumLNAAs) (*P* = 0.005) and branched chain amino acids (sumBCAAs) (*P* = 0.016) were reduced in the CD group (Fig. 2; Table 3). Specifically, tyrosine (*P* = 0.048), phenylalanine (*P* = 0.008), leucine (*P* = 0.031), and isoleucine (*P* = 0.003) were lower with a trending decrease in valine (*P* = 0.090). After correction for sumLNAA concentration, tyrosine (marker of dopamine synthesis) remained lower in CD (*P* = 0.048), but tryptophan (marker of serotonin synthesis) was not different between the groups. Whole body production (WBP) of the individual LNAAs and the conversion of phenylalanine to tyrosine, reflecting postabsorptive net protein breakdown, were not different between the groups (Fig. 2). Clearance rates of phenylalanine (ANCOVA, *P* = 0.003) and leucine (ANCOVA, *P* = 0.013) were increased in the CD group, with valine clearance tending to be lower (ANCOVA, *P* = 0.071).

**Table 3:** Large neutral amino acid plasma concentrations, whole body production rates, and clearance rates in COPD non-depressed and COPD depressed

	COPD non-depressed (CN) (n = 51)	COPD depressed (CD) (n = 27)	Estimated difference	ANCOVA *p value*
Plasma concentrations (μmol/L)				
Tryptophan	35.76 [33.64, 37.89]	35.24 [31.77, 38.71]	− 0.26 [− 2.79, 2.27]	*0.783*
Tryptophan corrected for large neutral amino acids			1.07 [− 3.39, 5.53]	*0.656*
Tyrosine	54.23 [50.65, 57.80]	47.11 [43.14, 51.08]	− 8.13 [− 13.63, − 2.63]	***0.005***
Tyrosine corrected for large neutral amino acids			− 5.58 [− 10.99, − 0.17]	***0.048***
Phenylalanine	48.54 [45.71, 51.38]	43.40 [40.55, 47.57]	− 5.75 [− 9.86, − 1.64]	***0.008***
Leucine	110.50 [98.87, 122.10]	95.95 [79.09, 106.70]	− 18.77 [− 34.54, − 2.99]	***0.031***
Isoleucine	65.79 [58.34, 73.23]	53.83 [41.92, 63.58]	− 15.11 [− 24.20, − 6.02]	***0.003***
Valine	177.60 [162.70, 192.40]	163.30 [137.20, 177.70]	− 20.67 [− 42.12, 0.80]	*0.090*
Sum branched chain amino acids	332.40 [306.40, 358.40]	294.70 [243.10, 327.90]	− 49.57 [− 86.83, − 12.32]	***0.016***
Sum large neutral amino acids	435.20 [406.50, 463.90]	385.20 [322.20, 430.30]	− 63.42 [− 104.03, − 22.79]	***0.005***
Whole body production rates (μmol/h)				
Phenylalanine	3507.33 [3250.40, 3764.27]	4022.20 [3522.65, 4521.75]	271.60 [− 286.95, 830.06]	*0.214*
Tyrosine	3188.99 [2894.67, 3483.31]	3618.15 [3059.27, 4177.04]	107.01 [− 514.88, 728.98]	*0.581*
Tryptophan	1175.83 [1026.55, 1325.11]	1326.93 [1088.30, 1565.57]	114.50 [− 145.50, 374.50]	0.382
Isoleucine	3673.94 [3094.86, 4253.01]	3989.06 [3155.51, 4822.61]	362.20 [− 623.90, 1348.00]	0.465
Valine	11,119.21 [9712.82, 12,525.61]	11,880.31 [9962.54, 11,602.38]	276.61 [− 1423.95, 1976.60]	*0.570*
Leucine	9782.64 [8789.57, 10,775.71]	10,352.46 [9102.55, 12,188.00]	− 3.91 [− 1384.21, 1376.47]	*0.811*
Phenylalanine to tyrosine (net protein breakdown)	216.80 [179.40, 235.00]	267.50 [200.50, 287.20]	34.51 [− 7.93, 76.96]	*0.104*
Branched chain amino acids	24,783.00 [21684.00, 27,881.00]	26,808.00 [20235.00, 31,134.00]	1354.79 [− 2141.15, 4851.15]	*0.315*
Clearance rates (L h^−1^)				
Leucine	1.98 [1.79, 2.18]	2.24 [1.78, 2.64]	0.34 [0.08, 0.60]	***0.013***
Valine	1.35 [1.24, 1.47]	1.47 [1.18, 1.76]	0.18 [0.004, 0.36]	*0.071*
Isoleucine	1.30 [1.09, 1.50]	1.51 [1.00, 1.74]	0.33 [− 0.08, 0.74]	*0.133*
Phenylalanine	1.55 [1.46, 1.64]	1.88 [1.51, 2.18]	0.30 [0.11, 0.49]	***0.003***
Tryptophan	0.72 [0.65, 0.80]	0.77 [0.62, 0.95]	0.06 [− 0.08, 0.21]	0.371

Values are mean [95% CI], except for when data was log-transformed for which geometric mean [95% CI] was used. LNAA: Large neutral amino acids. BCAA: Branched chain amino acids. WBP: Whole body production. Statistics are by ANCOVA with age and BMI as covariates, normal text is untransformed data and italicized is log-transformed data, bold is *P* < 0.05

#### Arginine and related amino acid metabolism

**Fig. 3:**
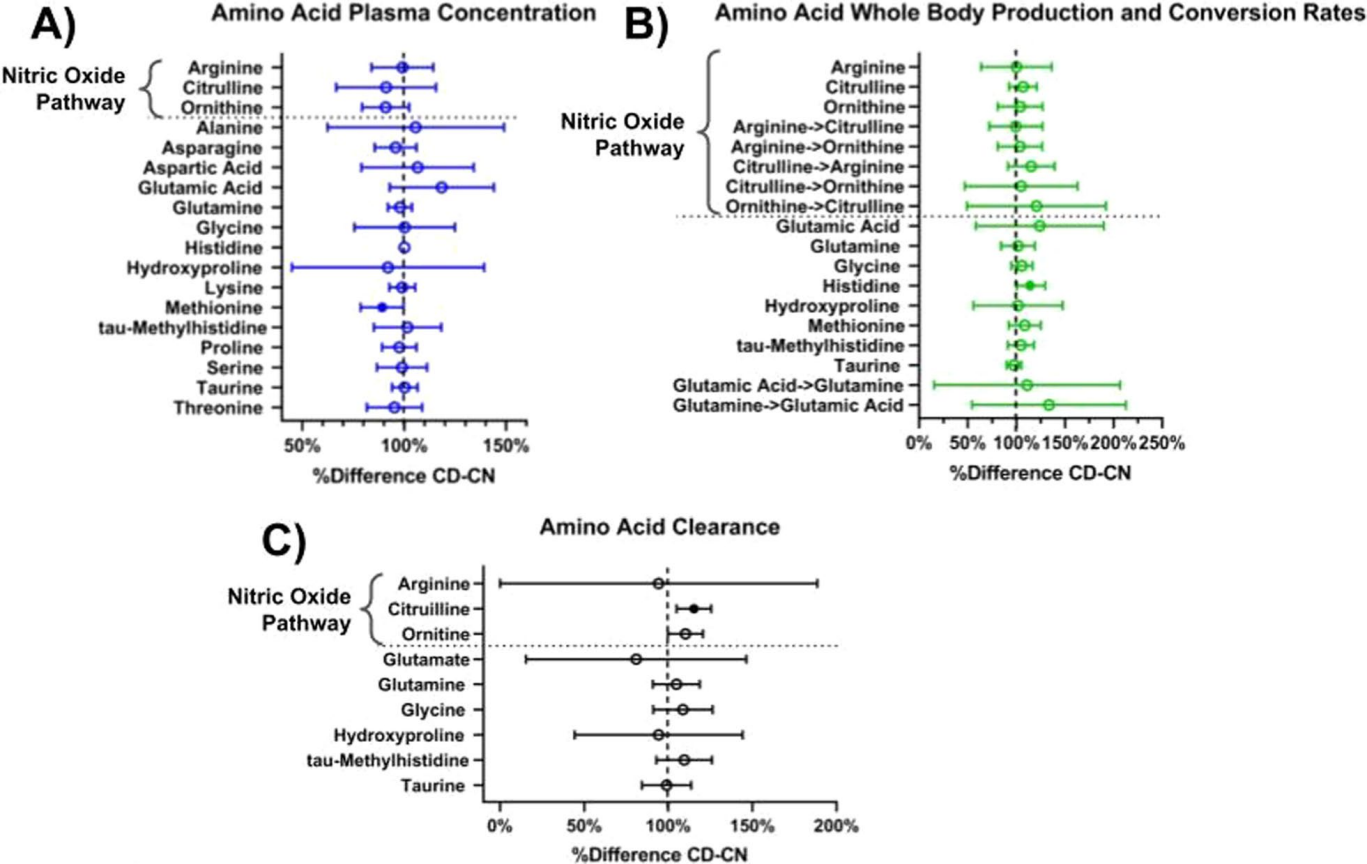
Differences in amino acid metabolism between non-depressed (CN) and depressed (CD) COPD patients. **A** Amino acid plasma concentration. **B** Amino acid whole body production and conversion rates. **C** Amino acid clearance rate. Percent difference of CD relative to CN. Mean ± 95% CI. Filled in circles are *P* < 0.05 by ANCOVA with age and BMI as covariates

Plasma concentrations (Fig. 3A; Table 4) or whole body production (Fig. 3B) of arginine, ornithine, and citrulline and conversion rates between arginine and citrulline (Fig. 3B) were not different between the CN and CD groups. While arginine clearance was not different between the groups (ANCOVA, *P* = 0.879), citrulline clearance was increased (ANCOVA, *P* = 0.006) and ornithine clearance tended to be increased (ANCOVA, *P* = 0.075) in the CD group (Fig. 3C).

**Table 4:** Arginine and related amino acids plasma concentrations, whole body production rates, and clearance rates in COPD non-depressed and COPD depressed

	COPD non-depressed (CN) (n = 51)	COPD depressed (CD) (n = 27)	Estimated difference (t-test)	ANCOVA *p value*
Plasma concentrations (μmol/L)				
Arginine	69.46 [62.83, 76.10]	68.22 [54.30, 75.72]	− 0.54 [10.00, − 11.08]	*0.943*
Citrulline	34.54 [31.00, 38.08]	31.12 [22.98, 37.61]	− 3.07 [− 11.54, 5.40]	*0.438*
Ornithine	55.70 [51.75, 59.66]	51.89 [44.19, 63.77]	− 5.066 [− 11.49, 1.35]	*0.140*
Whole body production rates (μmol/h)				
Arginine	9405.40 [7503.03, 11,307.80]	9394.86 [8228.46, 10,561.30]	5.67 [− 3398.25, 3409.58]	*0.855*
Citrulline	1028.99 [950.60, 1107.42]	1124.34 [989.04, 1259.63]	70.86 [− 76.83, 218.60]	*0.334*
Ornithine	2031.62 [1856.21, 2207.03]	2277.54 [1954.20, 2600.88]	81.64 [− 386.24, 549.71]	*0.544*
Citrulline to arginine (ARG de novo production)	1075.75 [951.58, 1199.93]	1311.09 [1072.80, 1549.37]	164.97 [− 93.01, 422.81]	*0.224*
Arginine to citrulline (NO production)	389.80 [154.50, 625.20]	372.80 [82.24, 247.60]	− 1.67 [− 108.32, 105.00]	*0.974*
Arginine to ornithine	2258.60 [1631.89, 2885.32]	2706.34 [1709.68, 3702.99]	85.26 [602.25, − 431.73]	*0.819*
Ornithine to citrulline	116.50 [97.74, 135.20]	145.00 [118.10, 187.70]	24.12 [− 59.20, 107.42]	*0.555*
Citrulline to ornithine	292.80 [195.40, 390.10]	364.30 [206.2, 522.30]	14.57 [184.66, − 155.53]	*0.902*
Clearance rates (L h^−1^)				
Arginine	3.00 [2.51, 3.49]	2.843 [2.52, 3.34]	− 0.172 [2.66, − 3.00]	*0.879*
Citrulline	0.67 [0.62, 0.71]	0.75 [0.66, 0.80]	0.10 [0.03, 0.17]	***0.006***
Ornithine	0.79 [0.73, 0.84]	0.86 [0.71, 1.00]	0.08 [− 0.002, 0.16]	*0.075*

Values are mean [95% CI], except for when data was log-transformed for which geometric mean [95% CI] was used. DLCO: diffusing capacity for carbon monoxide. WBP: Whole body production. Statistics are by ANCOVA with age and BMI as covariates, normal text is untransformed data and italicized is log-transformed data, bold is *P* < 0.05

#### Remaining amino acid metabolism

Comparable plasma concentrations, whole body production and clearance rates (Fig. 3A–C; Additional file 1: Table S3, Additional file 1: Table S4, Additional file 1: Table S5) were found for the remaining amino acids except for a lower plasma concentration of methionine (ANCOVA, *P* = 0.048) (Fig. 3A) and increased production of histidine (ANCOVA, *P* = 0.044) (Fig. 3B) in the CD group.

We also interrogated whether inclusion of antidepressant use, SSRI use, SNRI use, and anxiolytic use as covariates in our model would alter results and doing so did not alter our findings. In line with this, we found comparable significant findings when analyzing only subjects who used antidepressants in CN and CD groups (Additional file 1: Table S6, Additional file 1: Table S7, Additional file 1: Table S8, Additional file 1: Table S9, Additional file 1: Table S10), though sample sizes were smaller (CN n = 23, CD n = 15).

## Discussion

In this present study, we examined whether COPD patients with depressive symptoms were characterized by a specific clinical, functional and metabolic phenotype. While there were no differences in lung function and disease history between the depressed (CD) and non-depressed (CN) COPD groups, the impact of COPD on quality of life was higher in the CD group. Furthermore, the CD patients were on average obese, and were characterized by shorter 6MWT distances, physical inactivity, and poor quality of life compared to the CN group. Metabolism of Large Neutral Amino Acids (LNAA) and particularly of the BCAA, was altered in the CD group.

For this study, 78 COPD outpatients were randomly recruited from the community. Prevalence of depression based on a depression score ≥ 8 was 34.6% (27/78), which is in line with 33–57% of mild depression previously found (depression score: 8–11 or Geriatric Depression Scale score ≥ 11/30) [2–4, 6]. The depressed and non-depressed groups were of a similar age range (46–76 years) and had comparable lung function characteristics, as previously observed [13–16, 18, 65]. Although elevated CRP levels are associated with depression [66], remarkably lower CRP values were found in the studied CD compared to CN, though both groups had an elevated systemic inflammatory response (CRP levels > 3 mg/L) [67]. Moreover, we observed no difference in the proportion of patients using antidepressants/anxiolytics between non-depressed COPD and those experiencing depression (48% CN vs. 59% CD), suggesting there may be a subpopulation that either are non-adherent for medication use for reasons such as fear of side-effects and stigma [68–70] or has treatment-resistant depression.

The present study examined whether disturbances in amino acid metabolism may play a role in the etiology of depression in COPD, as this information may aid in identifying specific nutritional targets of treatment. As multiple other factors may affect amino acid metabolism, we first examined whether our depressed subjects were characterized by certain daily functional, body composition, and lifestyle features.

### Physical muscle function, body composition, cognitive function, and quality of life

We found a slight lower 6-min walk distance but not in muscle strength in the depressed COPD patients [9, 16, 25, 71, 72], which might be explained by the lower daily physical activity level. In line, a 6-week exercise regimen previously found to lead to improved depressive symptoms [13]. Depression in COPD was not associated with changes in BMI or body composition in line with previous studies [9, 16, 18], though one study did identify higher levels of depression in underweight COPD patients [15] and others found an association with increased BMI [13, 14]. However, depression was associated with a reported lower quality of life and elevated CAT scores, consistent with previous studies regarding mildly depressed COPD patients [13, 15, 16, 18, 72]. Additionally, while CD on average had mild cognitive impairment (MoCA score of 24.9), there was no significant difference in cognitive function as assessed by STROOP and TMT tests between the CD and CN, consistent with one study showing only a trending association between cognitive impairment and depression [27] and another study finding only borderline significance for the copy drawing test, which assesses visuospatial and praxis skills [28]. This means if depression does impact cognitive function in COPD, it may be harder to discern due to the negative impact the presence of COPD already has on cognitive function.

### Large neutral amino acid metabolism

In the present study, we examined plasma amino acid profile and whole-body production rates of amino acids known to play a role in mood (e.g. LNAA, BCAA, and arginine). We observed a decrease in all LNAA plasma concentrations except for tryptophan in the patients with depression. This decrease is due to increased clearance of some of the LNAAs (leucine, valine, phenylalanine) without a compensatory increase in their whole body productions. Normally, a reduced tryptophan:LNAA ratio is associated with depression [35, 73–75], the hypothesis being that this reduced ratio represents increased competition for the L-type amino acid carrier that LNAAs use to cross the blood–brain barrier, decreasing tryptophan availability for serotonin production [76]. However, decreased LNAA levels and comparable values were found for adjusted tryptophan plasma concentrations in our depressed COPD group. Elevated serotonin plasma levels were found in COPD patients and associated with worse symptoms and increased number of exacerbations [77, 78], with one study positing serotonin as a contributor in the pathogenesis of COPD [79]. Thus the decreased competition of Trp with other LNAAs and subsequent functional increase in availability may explain in part the decreased quality of life and worse disease severity reported in our study population. More research is needed to examine whether there is an insensitivity to serotonin signaling in the brains of COPD patients because despite the suggested elevated circulating serotonin there is still a higher rate of depression.

Lower levels of BCAAs have previously been reported in individuals with COPD with low muscle mass [29, 80], however, fat-free mass of our studied CD group can be considered as normal. Reduced plasma BCAA concentrations have previously been proposed as a new biomarker for depression after identifying a negative correlation between BCAAs and depression scores [81]. Moreover, increased dietary intake of BCAAs were able to decrease the odds of depression in healthy adults [82], and depression scores improved after 4 months of leucine enriched nutritional supplementation in COPD [83].

One study revealed the underlying mechanism by which BCAAs impact mood by demonstrating that BCAAs supplementation promotes resilience in mice exposed to chronic defeat stress by increasing Brain Derived Neurotrophic Factor (BDNF)/TrkB (the receptor for BDNF) signaling in the hippocampus [84]. Exercise probably works via the same mechanism, promoting BDNF expression in the hippocampus [85], potentially revealing why a 6-week exercise regimen showed improved depressive symptoms [13]. Hallmarks of depression include decreased brain BDNF levels and TrkB signaling [86] as well as hippocampal degeneration [87]. Furthermore, decreased serum levels of irisin, a potent inducer of BDNF, is associated with increased mood disturbances in COPD [88] and hippocampal atrophy has been identified in COPD [89]. Increasing BDNF/TrkB signaling activates the mechanistic target of rapamycin complex 1 (mTORC1), which decreases depression in the brain [90]. Additionally, BCAAs, predominantly leucine, are capable of activating mTORC1 directly [91]. We hypothesize that mTORC1 activation in the hippocampus is taking a double hit due to lower BCAA levels identified in the CD group, indirectly by reduced stimulation of BDNF/TrkB signaling and directly by reduced stimulation of mTORC1 itself, resulting in the observed depressive symptoms. The lower levels of BCAAs that individuals with COPD have independent of muscle function and body composition may therefore contribute to increased risk of developing depression.

Revealing mTORC1 as a potential mediator of depression in COPD also suggests considering use of alternative antidepressants as opposed to serotonin reuptake inhibitors (SSRIs), which are the current first line treatment for depression in COPD patients. Rapid acting antidepressants that promote mTORC1, such as low dose ketamine and scopolamine [92, 93], may be more effective and beneficial in the COPD population. Recently, the FDA has approved esketamine, the s-enantiomer of ketamine for treatment-resistant depression [94]. This new class of antidepressants that target mTORC1 may potentially prove more effective in depressed COPD patients while also minimizing side effects associated with SSRI use. Further research is needed in this area.

### Arginine and related amino acid metabolism

We observed an increase in citrulline and ornithine clearance without other alterations in the arginine pathway in the CD group. Perturbations in the arginine pathway and metabolites have previously been associated with depression. Both human and animal studies have reported a link between dysregulation of plasma concentrations of arginine and related catabolic products and nitric oxide (NO) imbalance with pathophysiology of major depressive disorder (MDD) [38, 39, 95–98].

NO has been shown to modulate synthesis of monoamine neurotransmitters (norepinephrine, serotonin, dopamine) as well as influence the hypothalamus–pituitary–adrenal axis [99]. Our previous work comparing COPD patients to healthy controls found alterations in the arginine pathway [31, 33] and a higher NO production [31]. Our present data in CD only identified an increase in clearance of citrulline and ornithine, suggesting arginine metabolism and metabolites may not be underlying contributors to depression in COPD. Alternatively, our study predominantly recruited individuals with mild depression, experiencing depressive symptoms that may not reach the level of requirement for diagnosis of the previously studied MDD.

### Methionine metabolism

We observed a decrease in the methionine plasma concentration, which is a key player in one-carbon metabolism, another pathway linked to depression. Methionine is converted by methionine adenosyltransferase to *S*-adenosyl-methionine (SAMe), which acts as a universal methyl donor for various important biological processes [100], including synthesis of monoamine neurotransmitters [101, 102]. Moreover, the methionine cycle crucially interacts with the folate cycle, which is essential for the synthesis and regeneration of tetrahydrobiopterin [103], an important cofactor for enzymes that convert amino acids to monoamine neurotransmitters [104].

SAMe has been assessed in numerous trials over the years as a potential antidepressant [105], with some trials identifying SAMe as a potentially beneficial add-on in antidepressant therapy that enhances the effectiveness of the drug it is paired with [106]. If methionine levels are decreased in a particular patient population, it may create a predisposition for the development of depression. Our previous work has shown a decrease in methionine levels in COPD patients compared to healthy controls [29, 32], potentially explaining in part the increased risk of depression in this population. In the present study, decreased methionine concentration in the plasma with no compensatory elevation of methionine WBP was identified in depressed individuals with COPD. More studies are needed whether subsequent perturbations in one-carbon metabolism and neurotransmitter synthesis contribute to the depressive symptoms reported in these individuals.

### Limitations

Although 78 COPD subjects were studied, the results might not be applicable to the general COPD population. Patients were those who voluntarily agreed to be part of the clinical trial and come in for questionnaires and testing, potentially excluding those suffering from moderate to severe depression, as is suggested by the average depression score in the CD group. This study is not powered for sex differences though some studies have shown association between female sex and depression in COPD populations [9, 14]. Moreover, this study is not powered for age differences but we did initially see significantly lower age in the depressed group before controlling for age and BMI in order to complete the ANCOVA analysis. This initial finding is consistent with other studies [9, 65] and is a potential future direction as more are recruited for the clinical trial. Additionally, we attempted to answer the clinically important question of metabolomic differences underlying treatment-resistant depression in COPD by comparing antidepressant users of the CN and CD groups. While comparable findings were obtained, a larger sample size is still required to identify a distinguishing phenotype.

## Conclusion

In conclusion, the present study revealed a set of metabolic changes in depressed COPD that include perturbation of large neutral amino acids and specifically BCAAs. Further research is warranted to determine if these disturbances are causative or consequences of depression in COPD. Though supplemental nutrition enriched in leucine has been shown to improve depressive symptoms during pulmonary rehabilitation, it remains to be shown that BCAA supplementation is beneficial for treating depression in COPD. Moreover, if BCAA imbalances and subsequent mTOR disturbances in the brain prove to be contributing factors to depression in COPD, newer antidepressants targeting mTOR may prove more effective in the COPD population. Ultimately, identification of an underlying metabolic phenotype in depressed COPD patients is critical to provide insight into potential nutritional and therapeutic treatments.

## Supplementary Information

**Additional file 1:** Supplementary Tables.

## Data Availability

The datasets generated and analyzed during the current study are available from the corresponding author on reasonable request.

## Abbreviations

BCAA: Branched chain amino acids
BDNF: Brain Derived Neurotrophic Factor
CAT: COPD assessment test
COPD: Chronic obstructive pulmonary disease
CVD: Cardiovascular disease
FEV1: Forced expiratory volume in 1 s
FFM: Fat-free mass
FFMI: Fat-free mass index
FM: Fat mass
FVC: Forced vital capacity
hs-CRP: High-sensitivity C-reactive protein
LNAA: Large neutral amino acids
MCI: Mild cognitive impairment
MDD: Major depressive disorder
MoCA: Montreal Cognitive Assessment
mTORC1: Mechanistic target of rapamycin complex 1
NO: Nitric oxide
PASE: Physical Activity Scale for the Elderly Questionnaire
SAMe: *S*-Adenosyl-methionine
SCWTs: Stroop color-word tests
SGRQ: St. George Respiratory Questionnaire
TMT: Trail making test
QoL: Quality of life
WBP: Whole body production
6MWT: 6-Min walk test
