# Supplementary material for: Disturbances in branched-chain amino acid profile and poor daily functioning in mildly depressed chronic obstructive pulmonary disease patients
Source: BMC Pulm Med. 2021 Nov 7;21:351. doi: 10.1186/s12890-021-01719-9 (PMC8573879; doi:10.1186/s12890-021-01719-9)
Supplement: Supplementary file 1 — Additional file 1: Supplementary Tables. [file 12890_2021_1719_MOESM1_ESM.docx]

| Supplementary Table 1: Wellbeing, quality of life, and lifestyle of the COPD non-depressed and COPD depressed. | | | | |
| --- | --- | --- | --- | --- |
|  | COPD Non-Depressed  (CN)  (n=51) | COPD Depressed  (CD)  (n=27) | Estimated Difference | ANCOVA  *p value* |
| **Wellbeing and Quality of Life** | | | | |
| Depression | 4.14  [3.56, 4.71] | 9.19  [8.55, 9.83] | 5.08  [4.15, 6.01] | **<0.0001** |
| Anxiety | 5.51  [4.59, 6.43] | 7.93  [6.59, 9.26] | 2.59  [1.02, 4.17] | **0.002** |
| Profile of Mood: negative | 12.64  [6.55, 24.40] | 25.52  [15.91, 40.93] | 10.09  [-3.02, 23.20] | *0.123* |
| Profile of Mood: total score | 5.24  [-5.95, 16.42] | 21.27  [5.48, 37.07] | 15.84  [-3.38, 35.06] | 0.102 |
| St. George Questionnaire Total score | 44.54  [37.75, 52.57] | 61.13  [54.61, 68.42] | 14.69  [3.60, 25.79] | ***0.011*** |
| St. George Questionnaire Symptoms | 60.12  [52.65, 67.59] | 70.33  [64.35, 76.32] | 10.92  [-0.70, 22.54] | 0.065 |
| St. George Questionnaire Activity | 65.91  [58.38, 73.43] | 79.58  [71.63, 87.53] | 14.68  [2.34, 27.02] | **0.021** |
| St. George Questionnaire Impacts | 34.33  [27.68, 40.98] | 50.39  [41.18, 59.61] | 17.38  [6.02, 28.74] | **0.004** |
| **Lifestyle and Daily dietary intake** | | | | |
| PASE (score) | 151.20  [131.00, 171.50] | 119.30  [105.60, 133.00] | -33.57  [-63.98, -3.15] | **0.031** |
| Caloric Intake (kcal) | 1705.53  [1537.38, 1892.08] | 1842.76  [1547.02, 2195.03] | 134.05  [-260.35, 528.50] | *0.527* |
| Protein intake (g) | 76.01  [65.69,86.33] | 74.86  [59.40,90.31] | 1.58  [-14.67,17.82] | *0.754* |
| Protein intake per kg body weight (g/kg) | 0.88  [0.77, 1.01] | 0.81  [0.63, 1.05] | -0.01  [-0.62, 0.59] | *0.813* |
| Fat intake (g) | 68.01  [57.65, 80.23] | 75.09  [60.97, 92.48] | 4.366  [-9.50, 18.24] | *0.548* |
| Carbohydrate intake (g) | 163.60  [138.60, 193.20] | 215.50  [181.60, 255.60] | 43.90  [3.44, 84.36] | ***0.043*** |
| Values are mean [95% CI], except for when data was log-transformed for which geometric mean [95% CI] was used. Hospital Anxiety and Depression Scale and Profile of Mood States (POMS) Questionnaire were used to quantify depression, anxiety, and negative and total mood states. Physical Activity Scale for the Elderly questionnaire (PASE). Statistics are by ANCOVA with age and BMI as covariates. Normal text is untransformed data and *italicized* is log-transformed data, **bold** is p < 0.05. | | | | |

| Supplementary Table 2: Body composition, muscle function, and cognitive measurements of the COPD non-depressed and COPD depressed. | | | | |
| --- | --- | --- | --- | --- |
|  | COPD Non-Depressed  (CN)  (n=51) | COPD Depressed  (CD)  (n=27) | Estimated Difference | ANCOVA  *p value* |
| **Body Composition** | | | | |
| Lean mass (g) | 47233.97  [44124.49, 50562.58] | 49190.69  [44258.58, 54672.43] | -789.78  [-4204.31, 2623.60] | *0.459* |
| %Fat | 35.39  [32.84, 37.95] | 36.38  [32.65,40.11] | -1.20  [-4.44, 2.04] | 0.681 |
| FFM index (kg/m2) | 17.19  [16.39, 17.99] | 18.24  [16.78, 19.69] | 0.06  [-0.79, 0.91] | 0.163 |
| Lean mass per kg bw (%) | 48.60  [45.28, 51.93] | 50.84  [45.78, 55.90] | -0.79  [-4.20, 2.63] | *0.862* |
| Fat % android/gynoid (ratio) | 1.01  [0.95, 1.07] | 1.03  [0.92, 1.15] | -0.03  [-0.11, 0.06] | 0.587 |
| Visceral Adipose Tissue (g)  (CN=49, CD=27) | 907.10  [796.70, 1017.00] | 971.00  [784.70, 1157.00] | -47.41  [-174.80, 79.95] | 0.444 |
| **Physical Function** | | | | |
| Maximal handgrip strength (N) | 219.00  [200.80,237.10] | 212.10  [181.40,242.80] | -9.94  [-36.85, 16.99] | *0.467* |
| % total handgrip strength lost | 20.48  [16.81,24.15] | 25.63  [19.62,31.64] | 5.24  [-1.49, 11.97] | 0.125 |
| Peak torque leg extension (Nm) | 78.98  [68.97,88.98] | 81.04  [68.55,93.52] | -1.97  [-21.43, 17.50] | *0.880* |
| Maximal leg extension force per kg fat-free mass (N/kg) | 4.85  [4.49,5.22] | 4.81  [4.30,5.32] | -0.04  [-0.66, 0.57] | 0.896 |
| Leg Strength lost (fatigue) | 0.35  [0.25,0.45] | 0.38  [0.21,0.56] |  | *0.968* |
| Usual gait speed (m/sec) | 1.01  [0.92,1.10] | 0.91  [0.75,1.08] | -0.09  [-0.26, 0.07] | 0.270 |
| Fast gait speed (m/sec) | 1.44  [1.32,1.56] | 1.32  [1.09,1.55] | -0.12  [-0.34, 0.10] | 0.278 |
| 6 Minute Walk Distance (m) | 361.50  [332.80,390.20] | 309.20  [232.80,385.60] | -53.40  [-118.40, 11.57] | *0.055* |
| **Cognition** | | | | |
| TMT part A | 36.52  [31.55,41.49] | 36.10  [31.74,40.47] | -0.48  [3.01, -3.97] | *0.785* |
| TMT part B | 81.46  [70.22,92.69] | 86.10  [68.03,104.20] | 5.81  [-16.01, 27.62] | *0.586* |
| Stroop part 1 | 58.90  [53.35,64.45] | 59.71  [54.33,65.09] | 1.72  [-3.14, 6.59] | *0.516* |
| Stroop part 2 | 73.00  [68.12,77.87] | 72.32  [65.94,78.70] | -0.34  [-11.12, 10.44] | *0.941* |
| Stroop part 3 | 124.30  [112.20,136.50] | 128.20  [115.80,140.60] | 5.84  [-5.34, 17.02] | *0.339* |
| MoCa test | 26.19  [25.34, 27.04] | 24.88  [23.63, 26.12] | -1.26  [-2.78, 0.26] | 0.101 |
| Values are mean [95% CI], except for when data was log-transformed for which geometric mean [95% CI] was used. ‘%total handgrip strength lost’ and ‘Leg Strength lost’ are average force output of the first 3 repetitions minus the last 3 repetitions divided by average force output of the first 3 repetitions. Statistics are by ANCOVA with age and BMI as covariates, except for Body Composition measurements for which ANCOVA only used age as a covariate. Normal text is untransformed data and *italicized* is log-transformed data, **bold** is p < 0.05. | | | | |

| Supplementary Table 3: Remaining amino acid plasma concentrations in COPD non-depressed and COPD depressed. | | | | |
| --- | --- | --- | --- | --- |
|  | COPD Non-Depressed  (CN)  (n=51) | COPD Depressed  (CD)  (n=27) | Estimated Difference  (t-test) | ANCOVA  *p value* |
| ***Plasma concentrations (μmol/L)*** | | | | |
| Aspartic Acid | 1.88  [1.56, 2.20] | 2.07  [1.59, 2.55] | 0.13  [-0.39, 0.64] | *0.589* |
| Glutamic Acid | 48.58  [41.00, 56.16 ] | 60.45  [46.51, 74.38] | 8.98  [-3.42, 21.38] | *0.123* |
| Hydroxyproline | 13.71  [11.58, 15.85] | 12.97  [10.94, 15.01] | -1.09  [-7.55, 5.38] | *0.810* |
| Asparagine | 59.14  [55.71, 62.57] | 56.84  [52.36, 61.32] | -2.48  [-8.56, 3.60] | *0.431* |
| Glutamine | 570.90  [545.20, 596.60] | 548.70  [506.50, 590.90] | -11.85  [-45.61, 21.91] | *0.426* |
| Serine | 71.30  [66.34, 76.27] | 70.27  [62.91, 77.64] | -0.76  [-9.55, 8.04] | 0.864 |
| Glycine | 234.60  [214.50, 254.70] | 229.20  [195.60, 262.80] | 0.61  [-57.31, 58.52] | *0.870* |
| Threonine | 112.80  [103.90, 121.70] | 108.00  [95.38, 120.50] | -5.32  [-20.58, 9.95] | *0.493* |
| Alanine | 318.60  [293.50, 343.80] | 338.40  [282.10, 394.60] | 18.13  [-119.85, 156.16] | *0.739* |
| Taurine | 38.02  [35.12, 40.91] | 37.78  [34.24, 41.33] | 0.11  [-2.27, 2.49] | *0.928* |
| Proline | 165.50  [151.00, 180.00] | 171.10  [139.30, 202.80] | -3.91  [-17.89, 10.07] | *0.665* |
| tau-Methylhistidine | 5.10  [4.35, 5.86] | 5.49  [4.37, 6.62] | 0.09  [-0.76, 0.93] | *0.713* |
| Methionine | 19.82  [18.56, 21.08] | 17.74  [16.56, 18.93] | -2.12  [-4.23, -0.01] | ***0.048*** |
| Histidine | 60.41  [57.08, 63.73] | 60.02  [57.01, 63.04] | 0.06  [-0.43, 0.56] | *0.791* |
| Lysine | 150.90  [139.70, 162.10] | 152.60  [141.90, 163.40] | -1.38  [8.21, -10.96] | *0.711* |
| Values are mean [95% CI], except for when data was log-transformed for which geometric mean [95% CI] was used. Statistics are by ANCOVA with age and BMI as covariates, normal text is untransformed data and *italicized* is log-transformed data, **bold** is p < 0.05. | | | | |

| Supplementary Table 4: Remaining amino acid whole body production rates in COPD non-depressed and COPD depressed. | | | | |
| --- | --- | --- | --- | --- |
|  | COPD Non-Depressed  (CN)  (n=51) | COPD Depressed  (CD)  (n=27) | Estimated Difference  (t-test) | ANCOVA  *p value* |
| ***Whole body production rates (μmol/h)*** | | | | |
| Methionine | 1774.91  [1589.26, 1960.57] | 2063.57  [1764.65, 2362.49] | 153.32  [-137.98, 444.55] | *0.217* |
| Glutamic Acid | 44089.65  [37777.67, 50401.62] | 59168.83  [37146.98, 81190.67] | 10586.61  [-18438.14, 39617.80] | *0.444* |
| Hydroxyproline | 622.01  [475.60, 768.40] | 749.80  [397.10, 1102.49] | 10.97  [-274.10, 296.05] | *0.791* |
| Glutamine | 35253.11  [32399.81, 38106.00] | 38456.90  [33087.87, 43825.92] | 583.32  [-5569.76, 6736.40] | 0.565 |
| Glycine | 16042.36  [14394.36, 17690.36] | 17359.62  [15460.60, 19258.63] | 913.63  [-867.14, 2694.75] | *0.292* |
| Taurine | 2460.55  [2289.35, 2631.75] | 2557.71  [2200.20, 2915.21] | -61.51  [-248.89, 125.94] | *0.656* |
| tau-Methylhistidine | 75.36  [57.43, 93.30] | 89.58  [68.54, 110.60] | 3.51  [-6.72, 13.75] | *0.360* |
| Histidine | 3716.15  [3413.99, 4018.31] | 4511.33  [3957.14, 5065.51] | 552.46  [1.49, 1106.42] | ***0.044*** |
| Glutamic Acid to Glutamine | 16527.58  [13140.30, 19914.86] | 19162.97  [12390.20, 25935.75] | 1826.12  [-13955.39, 17609.49] | *0.784* |
| Glutamine to Glutamic Acid | 36122.13  [27672.69, 44571.58] | 61126.40  [23496.40, 98756.40] | 12118.42  [-16434.86, 40678.75] | *0.390* |
| Values are mean [95% CI], except for when data was log-transformed for which geometric mean [95% CI] was used. WBP: Whole body production. Statistics are by ANCOVA with age and BMI as covariates, normal text is untransformed data and *italicized* is log-transformed data, **bold** is p < 0.05. | | | | |

| Supplementary Table 5: Remaining amino acid clearance rates in COPD non-depressed and COPD depressed. | | | | |
| --- | --- | --- | --- | --- |
|  | COPD Non-Depressed  (CN)  (n=51) | COPD Depressed  (CD)  (n=27) | Estimated Difference  (t-test) | ANCOVA  *p value* |
| ***Clearance rates (L·h^−1^ )*** | | | | |
| Glutamic Acid | 27.97  [19.58, 36.36] | 22.08  [15.31, 28.85] | -5.33  [-23.66, 12.98] | *0.484* |
| Hydroxyproline | 1.08  [0.72, 1.44] | 1.077  [0.79, 1.37] | -0.062  [-0.60, 0.48] | *0.694* |
| Glutamine | 1.32  [1.22, 1.43] | 1.40  [1.26, 1.56] | 0.06  [-0.11, 0.25] | 0.494 |
| Glycine | 1.54  [1.36, 1.72] | 1.69  [1.43, 1.94] | 0.134  [-0.14, 0.41] | *0.313* |
| Taurine | 1.43  [1.31, 1.54] | 1.41  [1.22, 1.61] | -0.02  [-0.22, 0.19] | 0.890 |
| tau-Methylhistidine | 0.30  [0.28, 0.33] | 0.33  [0.28, 0.38] | 0.03  [-0.02, 0.08] | 0.254 |
| Values are mean [95% CI], except for when data was log-transformed for which geometric mean [95% CI] was used. Clpa: Plasma clearance. Statistics are by ANCOVA with age and BMI as covariates, normal text is untransformed data and *italicized* is log-transformed data, **bold** is p < 0.05. | | | | |

| Supplementary Table 6: Large Neutral Amino Acid plasma concentrations, whole body production rates, and clearance rates in COPD non-depressed and COPD depressed using antidepressants. | | | | |
| --- | --- | --- | --- | --- |
|  | COPD Non-Depressed  (CN)  (n=23) | COPD Depressed  (CD)  (n=15) | Estimated Difference | ANCOVA  *p value* |
| ***Plasma concentrations (μmol/L)*** | | | | |
| Tryptophan | 39.80  [37.12, 42.48] | 35.98  [30.03, 41.93] | -4.10  [-8.93, 0.73] | *0.096* |
| Tryptophan corrected for Large Neutral Amino Acids |  |  | -2.62  [-7.15, 1.91] | *0.265* |
| Tyrosine | 60.18  [54.32, 66.03] | 48.78  [42.51, 55.05] | -13.30  [-21.21, -5.39] | ***0.002*** |
| Tyrosine corrected for Large Neutral Amino Acids |  |  | -9.20  [-16.50, -1.91] | ***0.017*** |
| Phenylalanine | 50.01  [45.53, 54.49] | 42.48  [37.59, 47.37] | -9.30  [-15.32, -3.29] | ***0.004*** |
| Leucine | 106.4  [93.97, 118.7] | 98.94  [82.29, 115.6] | -12.80  [-30.71, 5.11] | *0.186* |
| Isoleucine | 63.42  [56.11, 70.73] | 57.06  [46.89, 67.23] | -9.13  [-19.11, 0.85] | *0.082* |
| Valine | 175.50  [156.6, 194.5] | 171.90  [146.10, 197.60] | -13.04  [-40.68, 14.60] | *0.419* |
| sum Branched Chain Amino Acids | 328.80  [292.60, 365.10] | 301.90  [253.80, 350.10] | -42.40  [-94.27, 9.46] | *0.132* |
| sum Large Neutral Amino Acids | 439.00  [396.70, 481.40] | 393.20  [340.20, 446.20] | -64.88  [-124.39, -5.33] | ***0.044*** |
| ***Whole body production rates (μmol/h)*** | | | | |
| Phenylalanine | 3713.21  [3292.81, 4133.61] | 4180.02  [3433.76, 4926.28] | 277.93  [-784.76, 1340.84] | *0.517* |
| Tyrosine | 3629.05  [3082.10, 4175.99 | 3692.90  [3024.43, 4361.38] | -190.35  [-917.06, 536.56] | *0.664* |
| Tryptophan | 1227.80  [950.97, 1504.62] | 1449.41  [1074.71, 1824.10] | 142.78  [-210.27, 495.74] | *0.394* |
| Isoleucine | 3511.54  [2558.84, 4464.23] | 4447.92  [3460.37, 5435.47] | 1226.99  [-86.42, 2540.40] | *0.074* |
| Valine | 11110.79  [9074.48, 13147.10] | 13206.79  [10316.20, 16097.38] | 1429.71  [-1813.18, 4672.59] | *0.327* |
| Leucine | 9910.00  [8656.36, 11163.64] | 10571.17  [8548.36, 12593.98] | 120.19  [-2051.59, 2292.19] | *0.849* |
| Phenylalanine to tyrosine (net protein breakdown) | 244.12  [197.87, 290.37] | 267.38  [208.57, 326.19] | 9.38  [-41.80, 60.57] | *0.647* |
| Branched Chain Amino Acids | 24841.73  [20498.62, 29184.85] | 29226.20  [23990.66, 34461.74] | 3550.79  [-2669.57, 9768.81] | *0.229* |
| ***Clearance rates (L·h^−1^ )*** | | | | |
| Leucine | 2.04  [1.73, 2.35] | 2.16  [1.81, 2.50] | 0.31  [-0.06, 0.69] | *0.122* |
| Valine | 1.31  [1.15, 1.47] | 1.45  [1.20, 1.70] | 0.22  [-0.07, 0.51] | *0.158* |
| Isoleucine | 1.16  [0.86, 1.47] | 1.64  [0.89, 2.38] | 0.84  [0.11, 1.58] | ***0.033*** |
| Phenylalanine | 1.54  [1.40, 1.69] | 1.86  [1.60, 2.12] | 0.37  [0.13, 0.60] | ***0.004*** |
| Tryptophan | 0.63  [0.52, 0.74] | 0.80  [0.61, 1.00] | 0.19  [-0.06, 0.43] | *0.137* |
| Values are mean [95% CI], except for when data was log-transformed for which geometric mean [95% CI] was used. LNAA: Large neutral amino acids. BCAA: Branched chain amino acids. WBP: Whole body production. Statistics are by ANCOVA with age and BMI as covariates, normal text is untransformed data and *italicized* is log-transformed data, **bold** is p < 0.05. | | | | |

| Supplementary Table 7: Arginine and related amino acids plasma concentrations, whole body production rates, and clearance rates in COPD non-depressed and COPD depressed using antidepressants. | | | | |
| --- | --- | --- | --- | --- |
|  | COPD Non-Depressed  (CN)  (n=23) | COPD Depressed  (CD)  (n=15) | Estimated Difference | ANCOVA  *p value* |
| ***Plasma concentrations (μmol/L)*** | | | | |
| Arginine | 73.44  [61.49, 85.39] | 74.12  [64.48, 83.75] | 0.60  [-2.35, 3.55] | *0.686* |
| Citrulline | 36.49  [31.16, 41.81] | 29.91  [25.41, 34.42] | -6.42  [-15.49, 2.66] | *0.145* |
| Ornithine | 56.34  [50.65, 62.03] | 55.29  [46.16, 64.43] | -2.79  [-10.15, 4.56] | *0.490* |
| ***Whole body production rates (μmol/h)*** | | | | |
| Arginine | 8891.02  [7495.10, 10286.79] | 9914.86  [8128.69, 11701.23] | 652.52  [-1182.08,2487.30] | *0.424* |
| Citrulline | 1071.20  [959.48, 1182.92] | 1114.23  [912.96, 1315.51] | 51.77  [-197.23,300.77] | *0.707* |
| Ornithine | 2130.47  [1863.84, 2397.10] | 2471.47  [1985.86, 2957.08] | 275.16  [-419.94, 970.52] | *0.359* |
| Citrulline to arginine (ARG *de novo* production) | 1147.33  [935.61, 1359.06] | 1336.05  [984.41, 1687.68] | 246.40  [-126.60, 619.40] | *0.224* |
| Arginine to citrulline (NO production) | 490.35  [43.26, 937.44] | 362.33  [-99.62, 824.28] | -304.26  [-1426.33, 818.29] | *0.641* |
| Arginine to ornithine | 2481.04  [1420.13, 3541.94] | 2596.53  [940.89, 4252.16] | -228.52  [-1091.23, 634.55] | *0.642* |
| Ornithine to citrulline | 112.56  [86.63, 138.48] | 148.70  [102.26, 195.14] | 29.65  [-112.79, 172.08] | *0.653* |
| Citrulline to ornithine | 329.54  [165.98, 493.09] | 326.49  [83.194, 569.78] | -27.45  [-160.08, 105.16] | *0.700* |
| ***Clearance rates (L·h^−1^ )*** | | | | |
| Arginine | 2.69  [2.31, 3.07] | 2.69  [2.33, 3.05] | 0.02  [-0.11, 0.14] | *0.800* |
| Citrulline | 0.63  [0.58, 0.69] | 0.74  [0.66, 0.82] | 0.14  [0.06, 0.22] | ***0.002*** |
| Ornithine | 0.77  [0.70, 0.85] | 0.82  [0.71, 0.93] | 0.09  [-0.04, 0.22] | *0.193* |
| Values are mean [95% CI], except for when data was log-transformed for which geometric mean [95% CI] was used. DLCO: diffusing capacity for carbon monoxide. WBP: Whole body production. Statistics are by ANCOVA with age and BMI as covariates, normal text is untransformed data and *italicized* is log-transformed data, **bold** is p < 0.05. | | | | |

| Supplementary Table 8 Remaining amino acid plasma concentrations in COPD non-depressed and COPD depressed using antidepressants. | | | | |
| --- | --- | --- | --- | --- |
|  | COPD Non-Depressed  (CN)  (n=23) | COPD Depressed  (CD)  (n=15) | Estimated Difference | ANCOVA  *p value* |
| ***Plasma concentrations (μmol/L)*** | | | | |
| Aspartic Acid | 2.04  [1.43, 2.66] | 2.21  [1.350, 3.08] | 0.03  [0.65, -0.59] | *0.959* |
| Glutamic Acid | 56.31  [44.62, 68.01] | 66.33  [41.65, 91.01] | 0.02  [-1.16, 1.20] | *0.885* |
| Hydroxyproline | 12.89  [10.18, 15.59] | 11.78  [9.410, 14.15] | -1.16  [-5.90, 3.58] | *0.610* |
| Asparagine | 57.97  [52.01, 63.93] | 58.46  [51.37, 65.55] | 0.18  [-8.81, 9.18] | *0.944* |
| Glutamine | 563.41  [521.44, 605.32] | 554.24  [501.21, 607.13] | -2.77  [-73.46, 67.90] | *0.911* |
| Serine | 70.58  [64.40, 76.76] | 73.06  [61.04, 85.08] | -0.73  [-4.82, 3.37] | *0.787* |
| Glycine | 239.09  [209.03, 269.28] | 234.04  [185.57, 282.53] | -1.87  [-69.21, 65.45] | *0.893* |
| Threonine | 114.72  [99.93, 129.51] | 118.87  [99.66, 138.13] | 3.26  [-25.77, 32.29] | *0.806* |
| Alanine | 318.83  [287.91, 349.69] | 376.62  [280.78 472.43] | 53.56  [-49.81, 156.95] | *0.280* |
| Taurine | 35.34  [30.60, 40.08] | 35.76  [31.00, 40.51] | 0.93  [-4.49, 6.36] | *0.745* |
| Proline | 170.50  [149.47, 191.54] | 182.81  [129.03, 236.70] | 2.32  [35.10, -30.47] | *0.920* |
| tau-Methylhistidine | 4.76  [4.14, 5.39] | 4.34  [3.37, 5.31] | -0.64  [-1.61, 0.34] | *0.222* |
| Methionine | 20.32  [18.07, 22.56] | 18.33  [16.56, 20.09] | -2.37  [-5.65, 0.92] | *0.159* |
| Histidine | 59.94  [54.47, 65.41] | 59.11  [55.61, 62.61] | -1.44  [-15.17, 12.30] | *0.874* |
| Lysine | 156.22  [135.52, 176.91] | 159.08  [141.31, 176.79] | 0.60  [-9.61, 10.81] | *0.837* |
| Values are mean [95% CI], except for when data was log-transformed for which geometric mean [95% CI] was used. Statistics are by ANCOVA with age and BMI as covariates, normal text is untransformed data and *italicized* is log-transformed data, **bold** is p < 0.05. | | | | |

| Supplementary Table 9: Remaining amino acid whole body production rates in COPD non-depressed and COPD depressed using antidepressants. | | | | |
| --- | --- | --- | --- | --- |
|  | COPD Non-Depressed  (CN)  (n=23) | COPD Depressed  (CD)  (n=15) | Estimated Difference | ANCOVA  *p value* |
| ***Whole body production rates (μmol/h)*** | | | | |
| Methionine | 1870.89  [1538.54, 2203.24] | 2115.29  [1695.80, 2534.78] | 97.12  [-433.82, 627.99] | *0.596* |
| Glutamic Acid | 50393.14  [39065.93, 61720.36] | 71023.26  [30842.13, 111204.4] | 19088.64  [-32582.54, 70759.81] | *0.412* |
| Hydroxyproline | 731.75  [444.15, 1019.40] | 654.62  [433.72, 875.53] | -132.86  [-584.03, 318.43] | *0.589* |
| Glutamine | 33484.97  [29363.49, 37606.44] | 38485.80  [32113.17, 44858.44] | 4319.70  [-5348.81, 13992.44] | 0.340 |
| Glycine | 17068.84  [14524.19, 19613.48] | 17342.97  [14766.67, 19919.28] | 248.54  [-1124.31, 1621.13] | *0.710* |
| Taurine | 2410.77  [2156.34, 2665.20] | 2677.60  [2135.39, 3219.81] | 123.23  [-430.58, 676.91] | *0.541* |
| tau-Methylhistidine | 69.70  [56.11, 83.29] | 80.46  [51.30, 109.6] | 0.41  [5.63, -4.81] | *0.956* |
| Histidine | 3849.62  [3415.63, 4283.62] | 4811.32  [4036.43, 5586.21] | 791.63  [-61.93, 1645.10] | *0.053* |
| Glutamic Acid to Glutamine | 17774.68  [11191.00, 24358.26] | 24134.85  [11515.65, 36753.85] | 6878.12  [-7462.53, 21218.76] | *0.347* |
| Glutamine to Glutamic Acid | 32795.23  [19110.08, 46480.38] | 81721.92  [7023.658, 156420.2] | 26886.57  [-17145.55, 70911.88] | *0.166* |
| Values are mean [95% CI], except for when data was log-transformed for which geometric mean [95% CI] was used. WBP: Whole body production. Statistics are by ANCOVA with age and BMI as covariates, normal text is untransformed data and *italicized* is log-transformed data, **bold** is p < 0.05. | | | | |

| Supplementary Table 10: Remaining amino acid clearance rates in COPD non-depressed and COPD depressed using antidepressants. | | | | |
| --- | --- | --- | --- | --- |
|  | COPD Non-Depressed  (CN)  (n=23) | COPD Depressed  (CD)  (n=15) | Estimated Difference | ANCOVA  *p value* |
| ***Clearance rates (L·h^−1^ )*** | | | | |
| Glutamic Acid | 29.84  [12.45, 47.22] | 24.24  [12.41, 36.08] | -2.29  [12.30, -16.87] | *0.789* |
| Hydroxyproline | 1.37  [0.58, 2.16] | 1.10  [0.73, 1.46] | -0.32  [-2.80, 2.16] | *0.815* |
| Glutamine | 1.25  [1.09, 1.42] | 1.35  [1.19, 1.51] | 0.12  [-0.11, 0.34] | *0.309* |
| Glycine | 1.59  [1.27, 1.91] | 1.63  [1.25, 2.02] | 0.08  [-0.39, 0.56] | *0.727* |
| Taurine | 1.45  [1.32, 1.58] | 1.54  [1.23, 1.85] | 0.03  [-0.36, 0.42] | *0.828* |
| tau-Methylhistidine | 0.31  [0.27, 0.35] | 0.35  [0.28, 0.42] | 0.04  [-0.05, 0.14] | *0.356* |
| Values are mean [95% CI], except for when data was log-transformed for which geometric mean [95% CI] was used. Clpa: Plasma clearance. Statistics are by ANCOVA with age and BMI as covariates, normal text is untransformed data and *italicized* is log-transformed data, **bold** is p < 0.05. | | | | |
